# Effects of Vegetation Cover on Community Structure of Rodents Based on Long Time Series from Dongting Lake, China

**DOI:** 10.3390/biology14070867

**Published:** 2025-07-17

**Authors:** Tian Huang, Yongcheng Tang, Yuwen Sun, Meiwen Zhang, Chen Zhang, Yunlin Zhao, Xiaoning Nan, Zhiyuan Hu, Zhenggang Xu

**Affiliations:** 1 Hunan Engineering Research Center of Ecological Environment Intelligent Monitoring and Disaster, Prevention and Mitigation Technology in Dongting Lake Region, College of Information and Electronic, Engineering, Hunan City University, Yiyang 413000, China; huangtian@hncu.edu.cn; 2 Hunan Research Center of Engineering Technology for Utilization of Environmental and Resources Plant, Central South University of Forestry and Technology, Changsha 410004, China; tyc2025010101@163.com (Y.T.);; 3 Dongting Lake Station for Wetland Ecosystem Research, Institute of Subtropical Agriculture, The Chinese Academy of Sciences, Changsha 410125, China; zhangmw@isa.ac.cn (M.Z.); zhangchenrosetiger@163.com (C.Z.); 4 College of Forestry, Northwest A&F University, Yangling 712100, China; 2023011663@nwafu.edu.cn (Y.S.); nxn@nwsuaf.edu.cn (X.N.)

**Keywords:** wildlife management, fatness, population density, NDVI, Microtus fortis, *Apodemus agrarius*

## Abstract

Rodents are the main wildlife species in the Dongting Lake area and can even cause significant biological disasters. In order to investigate the relationship between rodent communities and vegetation, the relationship between rodent population characteristics and vegetation cover between 2000 and 2020 was analyzed. The results showed that the rodent community in Dongting Lake has obvious spatial and temporal variability, and that *Microtus fortis* and *Apodemus agrarius* are currently the main rodent species in the area, with a higher population density in eastern Dongting Lake. Meanwhile, rodent fatness varied significantly between seasons, with the highest rodent fatness in winter at most survey sites. There was also significant spatial and temporal variability in vegetation cover, and the overall trend was increasing. There was a significant relationship between vegetation cover and rodent population density and fatness, and the relationship varied by rodent species. *Microtus fortis* population density was significantly correlated with medium to high levels of vegetation cover, but fatness was significantly correlated with low to medium levels of vegetation cover. The relationship between population density, fatness, and vegetation cover of *A. agrarius* is opposite to that of *M. fortis*. The study confirms that *M. fortis* is still the main pest rodent species in the Dongting Lake area, but the monitoring of *A. agrarius* and vegetation cover should be strengthened.

## Simple Summary

Rodents are important components of ecosystems with diverse ecological functions, both maintaining biodiversity and potentially causing disaster. Plants provide food and shelter for rodents and are closely associated with rodent community succession. Exploring the mechanisms of rodent community evolution is the theoretical basis for rodent integrative management. The study explored the relationship between vegetation cover and rodent community characteristics in Dongting Lake, China, based on long time series data. The study confirmed the significant spatial and temporal variability of rodent communities in the Dongting Lake area and the correlation with vegetation cover. The results of the study showed that there was a significant correlation between the fatness of *Microtus fortis* and low and medium levels of vegetation cover, while the relationship between the fatness of *Apodemus agrarius* and vegetation cover is the opposite. The study suggests that we should continue to further strengthen the long-term monitoring of rodent communities and vegetation communities in the Dongting Lake area. This study is the first time the relationship between rodent communities and vegetation cover in the Dongting Lake area has been monitored over a long period of time, which provides a reference for clarifying the patterns of animal succession and plant succession, and also provides a basis for wildlife management in the area.

## 1. Introduction

Rodents play an important role in the ecosystem as the most diverse species of mammals [1]. At the same time, rodents are also closely associated with humans, such as by destroying crops and spreading disease. Rodents are highly adaptable and widespread in a variety of ecosystems. Vegetation plays an important role in the maintenance of rodent community structure by not only providing important hiding places for rodents but also providing ample food sources for rodents. Studies have shown that the height, cover, density, and biomass of plants in ecosystems have important effects on population changes in rodent communities [2]. The distribution of rodent communities is dependent on the distribution of plant communities, with feedback effects between the structure and composition of the two ecosystem factors. Vegetation cover is a composite of plant density and growth conditions, and research has long focused on the effects of vegetation cover on rodent communities [3]. Studies in agroecosystems have shown that rodent densities peak during the maize lush period and that population densities decline after maize harvest [4]. In recent years, with the advancement of remote sensing technology, it has become an important trend to carry out large-scale vegetation cover monitoring [5]. A variety of different vegetation indices have been applied to assess the relationship between vegetation cover and rodents, and even to predict rodent densities and distributions [6,7]. The research in the habitats with different vegetation types in the Isimani landscape of Tanzania showed there was a strong positive correlation between rodent abundance and normalized difference vegetation index (NDVI) [7]. The above studies on the relationship between vegetation indices and rodent communities have focused mainly on farmland and forest ecosystems, with less attention paid to other ecosystems.

Lake beaches or the strand of rivers are often important habitats for rodents and are important areas for maintaining species diversity in the local region [8]. Dongting Lake is an important wetland in China and the world, and rodents are the main mammal community in the area [9,10]. The main rodent species in the area include *Microtus fortis*, *Apodemus agrarius*, *Rattus norvegicus*, *Rattus tanezumi*, *Mus musculus*, *Rattus losea*, *Niviventer confucianus*, *Niviventer fulvescens*, *Rattus nitidus*, and *Micromys minutus* [11]. Under the pressure of drastic water level change, the rodents in Dongting Lake beach would migrate back and forth between the beach and the nearby farmland and further cause rodent disasters (Figure S1). Obviously, Dongting Lake beach became an important source of rodents for the nearby ecosystems. *Microtus fortis* used to be the main rodent species in Dongting Lake beach and there have been many outbreaks of serious rodent disasters throughout history [12]. In addition to rodent feeding studies, current studies on rodents and plants in the Dongting Lake area are fragmented. In the Dongting Lake area, the structure of vegetation communities varies at different elevations and locations [13], and the structure of rodent communities also varies. Thus it is necessary to pay attention to the relationship between vegetation and rodent communities in the area.

The research group has long been concerned about the changes in rodent communities in the Dongting Lake area, and relevant studies have shown that the rodent communities have changed significantly, especially after the construction of the Three Gorges Project [10]. Recent changes in the rodent community are represented by the invasion of the *A. agrarius*, which used to live mainly on farmland, into the beach as one of the major rodent species [14]. Water level change is the most significant feature of Dongting Lake beach, and the Three Gorges Project directly affects it. The Three Gorges Project has also had a significant impact on the vegetation types of Dongting Lake beach. This study indicated that the wetland vegetation pattern significantly changed after the operation of the Three Gorges Project, mainly as a result of changes in submergence condition [15]. Based on previous studies, this study analyzed the relationship between vegetation changes and rodent communities in Dongting Lake based on long-term monitoring data. This study hypothesized that the spatial and temporal distribution characteristics of vegetation cover and rodents in the Dongting Lake area are closely related. This study will not only help to clarify the drivers of rodent community changes in the Dongting Lake wetland but will also provide a basis for rodent management in the area.

## 2. Study Area and Method

### 2.1. Study Area and Survey of Rodent Communities

**Figure 1:**
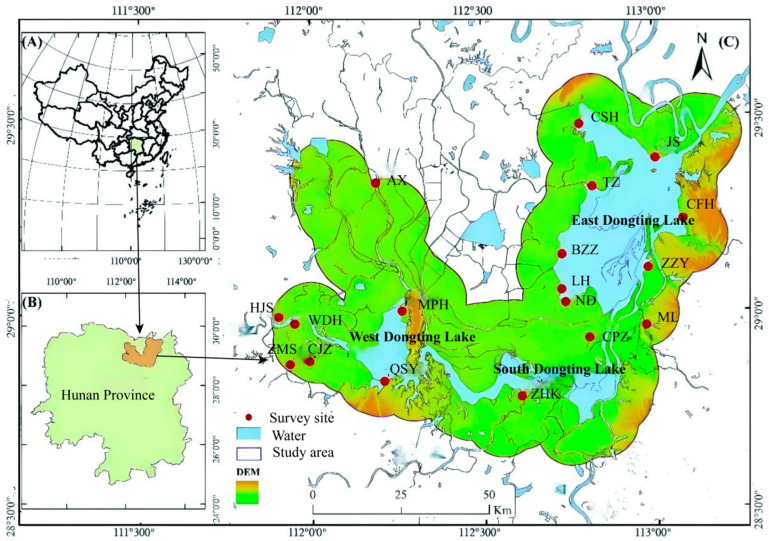
Study area and survey sites. (**A**) Location of Dongting Lake in China; (**B**) location of Dongting Lake in Hunan Province, China; (**C**) study area and survey sites. The abbreviations of survey sites are the initial letters of the Chinese names.

The Dongting Lake area is located on the south shore of the middle reaches of the Yangtze River (27°39′~29°51′ N, 111°19′~113°34′ E), China, and the wetland assumes various functions such as biodiversity protection and water resource supply, making it of great significance in guaranteeing the water ecological security of the middle and lower reaches of the Yangtze River (Figure 1). Dongting Lake is generally divided into three parts, namely East Dongting Lake, South Dongting Lake, and West Dongting Lake. The Dongting Lake wetland is rich in vegetation types, mainly including poplar communities, *Phragmites australis*, *Triarrhena lutarioriparia*, *Salix triandroides*, *Oenanthe javanica*, *Polygonum hydropiper*, *Carex brevicuspis*, and *Juncus bufonius* [16]. Since the study showed that the migratory distance of *M*. *fortis* in Dongting Lake does not exceed 10 km [12], a 10 km buffer of the Dongting Lake area was used as the study area (Figure 1).

During 2000–2020, the research group conducted long-term monitoring of rodent communities in the Dongting Lake area including 18 survey sites. Considering that poplar forest, reed community, and *Carex* spp. community are the main vegetation types in Dongting Lake beach (Figure S2), four sites, MPH, BZZ, LH, and CFH, which represent the above vegetation types, respectively, will be used as regular monitoring sites, while the remaining 14 survey sites will be used as temporary survey sites (Figure 1). The above rodent population survey sites cover the entire area of Dongting Lake evenly. The surveys were conducted quarterly. Rodent densities were surveyed using the trap method, and more details about the survey can be found in our previous study [9]. Then the population density (capture rate) and fatness degree were calculated [17]. The above investigation process was authorized by Dongting Lake Station for Wetland Ecosystem Research, Institute of Subtropical Agriculture, The Chinese Academy of Sciences and conducted under their supervision.

### 2.2. Vegetation Cover Monitoring Based on Remote Sensing

#### 2.2.1. Remote Sensing Data Acquisition and Processing

The image data used in this study is the MODIS 13Q1 dataset with spatial and temporal resolutions of 250 m and 16 d, respectively (https://ladsweb.modaps.eosdis.nasa.gov, accessed on 2 June 2025). A total of 480 images were downloaded from 2000 to 2020 and MODIS Reprojection Tool (MRT) software was use to preprocess the image, mainly including reprojection and format conversion [18]. Then the year-by-year time series NDVI data was obtained by the Maximum Value Compositing (MVC) method [19].

#### 2.2.2. Vegetation Cover Analysis

Since the remote sensing image of the Dongting Lake area is a mixed image element, where a single image element contains different proportions of land use types, Fractional Vegetation Cover (FVC) was employed to characterize vegetation cover [20]. The FVC was calculated as follows:

(1) $$FVC=\frac{NDVI-NDVIsoil}{NDVIveg-NDVIsoil}$$

FVC denotes the vegetation cover. NDVI_soil_ is the remote sensing information of pure soil pixels, and NDVI_veg_ is the remote sensing information of pure vegetation pixels. Referring to the results of other studies, the study set the cumulative percentage of 5% as NDVI_soil_ and the cumulative percentage of 95% as NDVI_veg_ [21]. The distribution maps of vegetation cover from 2000 to 2020 were finally obtained in the study area. At the same time, vegetation cover was categorized into five classes, namely low vegetation cover (0–0.3), medium–low vegetation cover (0.3–0.45), medium vegetation cover (0.45–0.6), medium–high vegetation cover (0.6–0.75), and high vegetation cover (0.75–1). The Theil–Sen Estimator and Mann–Kendall were employed to examine trends in vegetation cover [22]. The above trend analysis includes between years and also between seasons, and the researchers chose January, April, July, and October to represent the vegetation cover data in spring, summer, fall, and winter, respectively.

### 2.3. Data Statistical

The study used Pearson’s correlation analysis to statistically correlate vegetation cover with rodent community metrics with a significance criterion of 0.05. The Theil–Sen Estimator and Mann–Kendall analysis was performed in R 4.2. The remote sensing analysis and mapping used ENVI 5.0 and ArcGIS 10.0, respectively. The correlation analysis was performed in SPSS 18.0.

## 3. Results and Analysis

### 3.1. Characterization of Rodent Community Structure in Dongting Lake

#### 3.1.1. Spatial and Temporal Dynamics of Capture Rates

**Figure 2:**
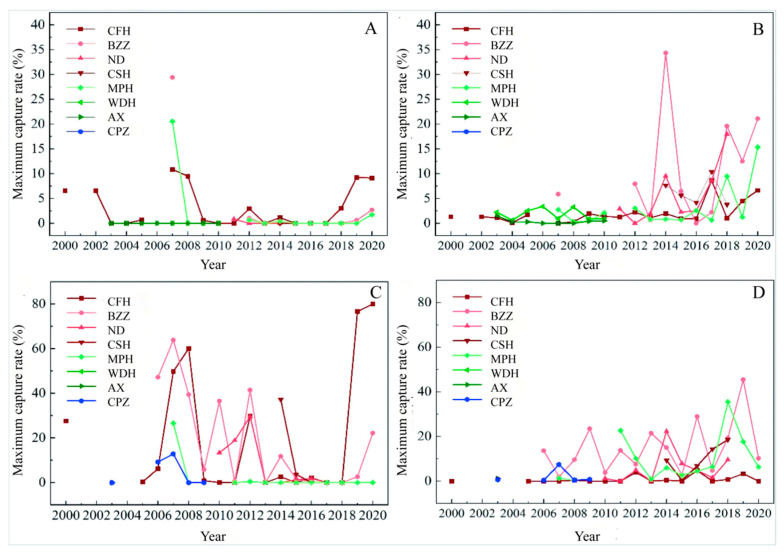
Temporal and spatial characteristics of capture rates of *M*. *fortis and A. agrarius*. (**A**) Maximum capture rate of *M*. *fortis* in wetland beach; (**B**) maximum capture rate of *A. agrarius* in wetland beach; (**C**) maximum capture rate of *M*. *fortis* in farmland; (**D**) maximum capture rate of *A. agrarius* in farmland.

During surveys of rodent populations, rodents were often not captured, which does not mean that the density was zero, but it was extremely low. Rodents captured during the survey mainly included eight species of *M. fortis*, *A. agrarius*, *R. norvegicus*, *M. minutes*, *N. confucianus*, *N. fulvescens*, *R. tanezumi*, and *R. losea*. Then the researchers chose three sites with relatively long survey time series and high rodent capture rates to count the maximum capture rate for each year (Figure S3), determining that *M*. *fortis* and *A. agrarius* were the dominant rodent species, which is consistent with our previous report [9]. Thus the follow-up analysis focused on the above rodent species. Of all the survey sites, eight of them contained more than five years of rodent density survey data and the above survey sites also covered almost all three parts of Dongting Lake. Since rodent disaster is mainly related to the maximum density [23], the maximum capture rate of *M*. *fortis* and *A. agrarius* at the above eight sites was further calculated for each year on the beaches and farmlands (Figure 2). Statistical results show that the population density of the main rodent species in Dongting Lake has typical spatial and temporal variability. From a regional perspective, the capture rate of East Dongting Lake was greater than that of West Dongting Lake and South Dongting Lake; from a habitat perspective, the capture rate of beaches was higher than that of farmland. The highest capture rate in beaches happened in 2019 (81.46%), dominated by *M. fortis*, which were captured 76.6% of the time. The highest capture rate in farmland occurred in 2007 (37.25%), and the capture rate of *M*. *fortis* was 29.41%. The population dynamics of different survey sites differed. Long-term low population density of *M*. *fortis* differed in MPH (West Dongting Lake) after an outbreak, while *M*. *fortis* were not captured in AX (West Dongting Lake) and WDH (West Dongting Lake) during the survey period. Capture rates varied among survey sites in East Dongting Lake during the survey period, with the largest fluctuations in CFH and BZZ. Population densities of *A. agrarius* show a fluctuating upward trend during the survey period.

#### 3.1.2. Fatness Characterization of Rodent Community in Dongting Lake

**Figure 3:**
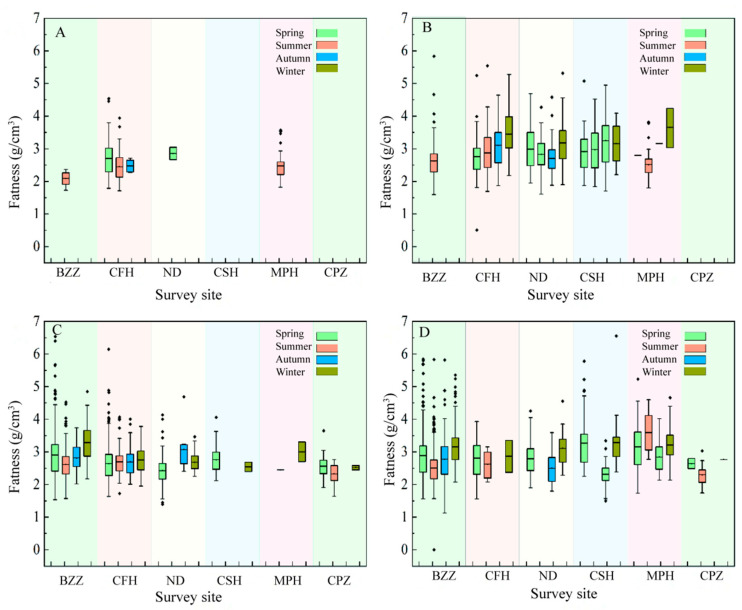
Characteristics of rodent fatness in different seasons at different sites. (**A**) Fatness of *M*. *fortis* in farmland; (**B**) fatness of *A. agrarius* in farmland; (**C**) fatness of *M. fortis* in in beach; (**D**) fatness of *A. agrarius* in beach.

Fatness is a comprehensive measure of an animal’s adaptation to its environment and has been widely used in rodent research [17]. Rodent fatness values directly affect their reproduction and can be used as an indicator to predict rodent population dynamics. Investigations showed that there were different characteristics of fatness between *Microtus fortis* and *A. agrarius* in Dongting Lake. Comparing the fatness of the *Microtus fortis* population and *A. agrarius* population in different habitats for more years, the fatness of *Microtus fortis* in farmland was slightly lower than that of *Microtus fortis* in the beach, and there was little difference between the fatness of *A. agrarius* in farmland and that of *A. agrarius* in beach. The fatness of *A. agrarius* in farmland was greater than that of *M*. *fortis* in farmland, and there was little difference between the fatness of *A. agrarius* in the beach and that of *Microtus fortis* in the beach (Figure S4). In terms of seasons, the variation in fatness varied from season to season. In general, most of the sites had higher fatness in winter than in other seasons (Figure 3).

### 3.2. Characteristics of Vegetation Cover Changes in Dongting Lake

#### 3.2.1. Interannual Spatial and Temporal Characteristics of Vegetation Cover

**Figure 4:**
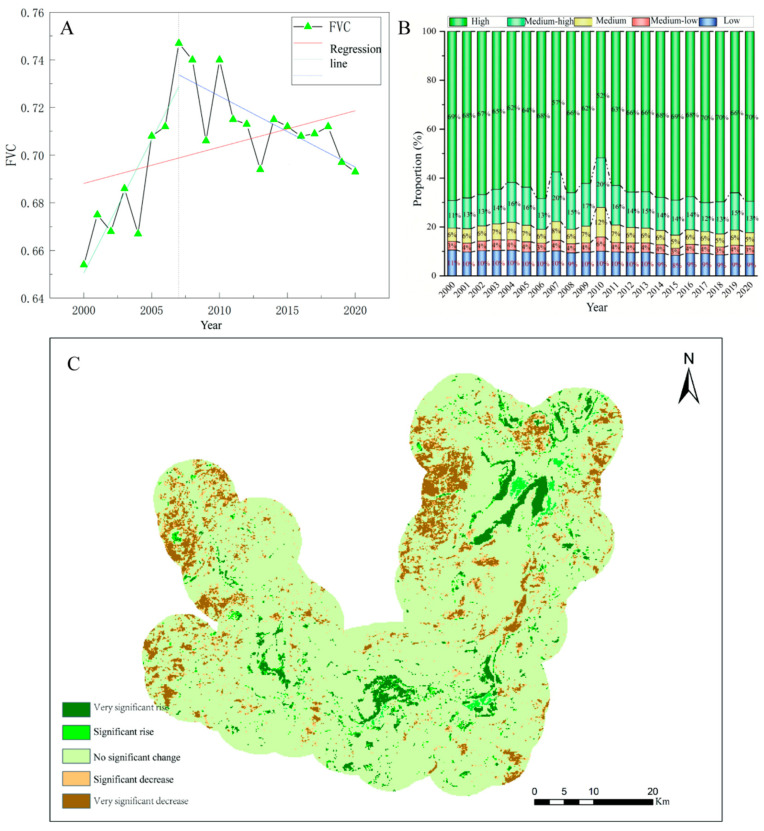
Interannual characterization of vegetation cover in Dongting Lake. (**A**) Overall trend of FVC in Dongting Lake; (**B**) changes in different levels of vegetation cover in Dongting Lake; (**C**) spatial distribution of FVC changes in Dongting Lake.

The study monitored the vegetation cover of Dongting Lake from 2000 to 2020. Generally speaking, the vegetation cover of the Dongting Lake area is good, with different characteristics in different areas, and there is a ring-like distribution of vegetation cover from the center to the periphery of the lake, from low to high (Figure S5). Overall, the vegetation cover in the study area showed an increasing trend from 2000 to 2020, with a significant oscillatory increase from 2000 to 2007, but an oscillatory decrease from 2007 to 2020 (Figure 4A). The vegetation cover in the Dongting Lake area was good, with the largest percentage of area covered by high vegetation cover, all exceeding 50%, and the smallest percentage of area covered by vegetation cover was that of medium–low vegetation cover, ranging from 3% to 6% (Figure 4B; Figure S6; Tables S1 and S2). For different levels of vegetation cover, low, and medium–low vegetation cover fluctuate less, while medium, medium–high, and high vegetation cover fluctuate more (Figure 4B). The study examined the vegetation cover change in each pixel over the 20-year period, and the results showed that most of the area had no-significant change in vegetation cover (81.57%), and that the areas with significant decrease (4.92%) and very significant decrease (7.88%) were larger than the areas with significant increase (1.42%) and very significant increase (4.21%). Spatially, there is a significant increase in the vegetation cover of the beach area and a decreasing trend in the vegetation cover of farmland (Figure 4C, Table S3).

#### 3.2.2. Seasonal Characteristics of Vegetation Cover

**Figure 5:**
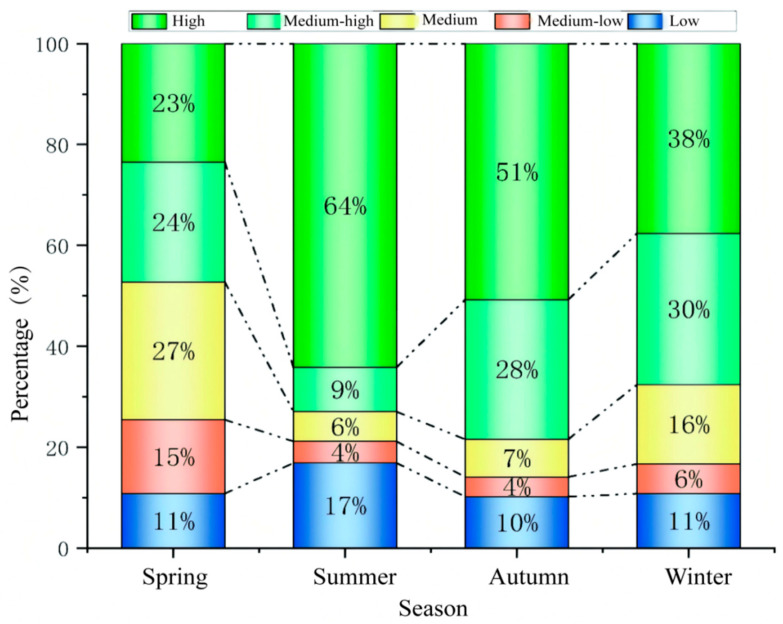
Proportion of vegetation cover at different levels in different seasons from 2000 to 2020.

The areas of different levels of vegetation cover varies in different seasons in the Dongting Lake area (Figure 5, Table S4). The highest percentage in the spring was medium vegetation cover (27.33%) and the smallest area percentage was low vegetation cover (10.8%). The percentage of high vegetation cover in summer is the highest percentage in the whole year, which is 64.17%; meanwhile, the percentage of low vegetation cover is also the highest percentage of in the whole year, which is 16.88%. The percentage of medium–low vegetation cover, medium vegetation cover, and medium–high vegetation cover area in summer is also the lowest in the four seasons, which is in line with the actual situation of Dongting Lake. In summer, the Dongting Lake basin enters the flood season, where the water level in the lake area is high and the inundated area is large, so the area proportion of low vegetation cover reaches the maximum of the whole year. At the same time, the rain and heat coincide with each other in summer, the plants enter into the vigorous growth period, and the area proportion of high vegetation cover is also the maximum of the whole year. In the autumn, with the significant decrease in water level, the percentage of low vegetation cover area decreased by 10.18% and the percentage of high vegetation cover area decreased to 50.73% compared to the summer, which may be related to agricultural production activities. Most of the farmland in the study area is paddy fields, mainly planted with rice, and the farmland is bare in the fall after the rice is matured and harvested, resulting in a decrease in vegetation cover. The area of high vegetation cover decreased further to 37.63% in winter compared to fall.

The spatial distribution of different levels of vegetation cover also varies considerably across seasons (Figure S7). In spring, high vegetation cover is mainly concentrated on the beach, while medium–low and medium vegetation cover is mostly found on farmland. Most of the lake beach is flooded in the summer, the water area is large, and farmland plants are growing vigorously, so the high vegetation cover area is also the largest of the year. In the fall, large areas of the Dongting Lake beach are exposed, and vegetation grows vigorously, with high vegetation cover in most areas of the lake beach. At this time, the vegetation cover of farmland is slightly lower than that in summer due to the harvesting of some crops (mainly rice). The areas with significant decreased vegetation cover were mainly concentrated in the farmland on West Dongting Lake, while the vegetation cover on most of the beach increased significantly. In winter, the vegetation cover declined further in fall, and the vegetation cover increased significantly in southern Dongting Lake and some farmland areas of eastern Dongting Lake; the area with a significant increase in the lake beach was located in eastern Dongting.

### 3.3. Relationship Between Vegetation Cover and Population Density

As the most serious area of rodent disaster in East Dongting Lake, and considering the continuity of the rodent survey data and the survey time, BZZ and CFH were chosen to analyze the relationship between vegetation cover and the population pattern of *M*. *fortis* and *A. agrarius* in the study (Table 1). There was a significant positive correlation between the population density of *M*. *fortis* and medium–high vegetation cover in BZZ, with a Pearson’s correlation coefficient of 0.502 (*p* < 0.05). *Microtus fortis* population density was negatively correlated with high vegetation cover and positively correlated with low, medium–low, and medium cover, but none were significant (*p* > 0.05). *Microtus fortis* population densities were negatively correlated with low, medium–low, and medium vegetation cover, and positively correlated with medium–high and high vegetation cover in CFH; statistical tests were not significant for either (*p* > 0.05). The relationship between population density and vegetation cover was consistent between the BZZ and CFH survey sites for *A. agrarius*, with a significant negative correlation (*p* < 0.05) between *A. agrarius* population density and low vegetation cover.

### 3.4. Relationship Between Vegetation Cover and Fatness

The relationship between population fatness of *M*. *fortis* and *A. agrarius* and vegetation cover varies among seasons (Table 2). The fatness of *M*. *fortis* was negatively correlated with low, medium–low, medium–medium, and medium–high vegetation cover at BZZ in spring, with a highly significant negative correlation with low vegetation cover (*p* < 0.01), and a non-significant positive correlation with high cover (*p* > 0.05). However, in summer, it showed the opposite relationship with spring. The fatness of *M*. *fortis* was highly significantly negatively correlated with low, medium, and medium–high vegetation cover in autumn (*p* < 0.01) and significantly positively correlated with high cover (*p* < 0.05). In winter, the fatness of *M*. *fortis* was not significantly negatively correlated with low and high cover (*p* > 0.05). The fatness of *A. agrarius* was negatively correlated with low, medium–low, medium, and medium–high vegetation cover at BZZ in summer (*p* > 0.05), and the correlation between fatness and vegetative cover was consistent in autumn and winter. At the CFH survey site, the fatness of *M*. *fortis* correlated consistently with vegetation cover, i.e., negatively but not significantly (*p* > 0.05) with low, medium–low, and medium vegetation cover, and non-significantly positively (*p* > 0.05) with medium–high, and high vegetation cover in spring and winter. In autumn, the fatness of *M*. *fortis* existed in a significant positive correlation with high vegetation cover (*p* < 0.05). In winter, the correlation between fatness of *A. agrarius* and vegetation cover were consistent with *M*. *fortis* fattening patterns, but there was a significant negative correlation between *A. agrarius* fatness and high vegetation cover in the spring (*p* < 0.05).

## 4. Discussion

Since *M*. *fortis* used to be a major pest rodent in the Dongting Lake area, previous studies have focused on this species and neglected the population of *A. agrarius*. In this study, the population characteristics of *M*. *fortis* and *A. agrarius* in the Dongting Lake area were investigated for the first time and their relationship with vegetation cover was analyzed. During the survey, fewer rodents were captured in West Dongting Lake, while rodents were mainly concentrated in East Dongting Lake. The reason may be related to the characteristics of Dongting Lake’s hydrological system, elevation, vegetation characteristics, and other factors [13]. The bed elevation of Dongting Lake is high in the west and low in the east [24], and East Dongting Lake is open, with a large area of plant communities favored by rodents. At the same time, the number of rodents in East Dongting fluctuates greatly because Dongting Lake joins Yangtze River at East Dongting Lake, which makes the East Dongting area subject to the greatest number and intensity of inundation by successive floods, and has the greatest impact on rodents that inhabit the area.

Fatness was highest in winter during the survey period at most sites, possibly related to female pregnancy or food resources. *Microtus fortis* were found to breed throughout the year, remaining highly fecund in the winter and lowest in the summer, while the summer was also the low point of breeding for *A. agrarius*, but in some poplar forest habitats, the breeding index was high [25]. The survey site of MPH in the study was dominated by poplar forest habitats, and it is possible that the high vegetation cover of poplar forests reduced the effects of high temperatures in summer on *A. agrarius*, resulting in increased fatness due to increased reproduction. At the same time, *A. agrarius* mainly feeds on plant seeds [26]. As the lake recedes in the autumn, *Carex* spp. and other plants begin to grow, so the lake beach has fewer seed resources for the rodents in the autumn, leading to the lowest fatness in the lake beach in the autumn. There is a correlation between rodent population density and fatness. Generally, rodents tend to have higher fatness when their population density is relatively low, while at relatively high population density, increased competitive pressures between individuals will lead to a decline in individual nutritional status and lower fatness [27]. High population densities and relatively low fatness in occurred the years 2007, 2008, and 2019 of the study, while low population densities and high fatness around 2015 and 2016 were consistent with the above pattern.

Yao Min et al. studied the ecological characteristics and distribution pattern of vegetation in the Dongting Lake area, and concluded that the vegetation was distributed in a mosaic in the area [28]. Lei Xuan et al. also found this pattern and concluded that the center of vegetation in Dongting Lake wetland has been migrating to the center of the lake in recent years [29]. The study further concluded that the operation of the Three Gorges Project has changed the hydrological characteristics of Dongting Lake, which in turn has changed the distribution of vegetation, with a decrease in the area of *Carex* spp. and an increase in the area of reeds and poplar forests [30], which may be an important reason for the decline and growth of the populations of *M*. *fortis* and *A. agrarius* in the lake area in recent years. The significant decrease in vegetation cover in some areas of the Dongting Lake beach during the study period may be related to the fact that the area had been planted with poplar on a large scale, which seriously damaged the integrity of the ecosystem, and was then vigorously removed, which was also confirmed by related studies [31]. The declining vegetation cover on farmland is mainly due to urbanization and changes in farming practices. In the past, Dongting Lake area mainly planted double-season rice, but due to the increase in planting cost, most of the area only planted single-season rice. The results of the study are consistent with the study of Han Qinzhe et al. which concluded that the overall vegetation cover in the Dongting Lake area showed a decreasing trend during the study period, and the decreasing areas were mainly concentrated in the paddy fields and towns [32]. The seasonal variation in vegetation cover in the Dongting Lake area is also closely related to the watershed area of Dongting Lake and local agricultural production activities, and the watershed area of the lake beach reaches the maximum throughout the year in summer, when the area of low vegetation cover is also the largest. Lake shore farmland has the least vegetation cover in the spring. During the survey, it was found that some of the paddy fields were dried up or planted with crops such as oilseed rape in the winter, which increased the vegetation cover in the winter to a certain extent, while the spring was the season of rice irrigation and sowing, so the lowest vegetation cover of lake shore farmland was in the spring. Du Bingxue et al. studied the relationship between plant area and water level in Dongting Lake from 2000 to 2014, and concluded that the plant area was large in the year of low water level; the intra-year variation in plant area showed a “bimodal type”, with the vegetation area being the largest in April, the smallest in July, and the second peak in November, which was in line with the results of the present study [33]. Overall, vegetation cover in Dongting Lake remained more closely related to *M*. *fortis* fatness, but high vegetation cover types were significantly correlated with *A. agrarius* fatness, which may be a result of the different biological characteristics of the two rodent species. *Microtus fortis* are still the main pest species in the Dongting Lake area, but it is important to continuously monitor changes in vegetation cover in the area, with particular attention being paid to small-scale variation in vegetation cover in order to inform sustainable rodent management.

There was a significant relationship between rodents and plants, but the relationship varied across rodent species. In this study, the relationship between the fatness of *M*. *fortiss* and *A. agrarius* and vegetation cover was completely different, which led to a simultaneous change in the composition of the rodent community in Dongting Lake with vegetation succession. Vegetation cover is a composite of plant species, plant growth conditions, and plant nutrition. Exploring the relationship between vegetation cover and rodent communities is of great significance for rodent management [3]. Vegetation cover and food availability are used to control rodent population densities in the management of rodent pests in and around maize fields [4]. Vegetation cover can be both a micro-ecological indicator and a macro-monitoring indicator transformed by remote sensing techniques [5,34]. We should emphasize the relationship between specific plants and specific rodents to clarify the details of rodent community changes, but we should also emphasize the long-term monitoring of macro-indicators. Habitat manipulation occurs necessarily through land use and intentionally to reduce shelter and food availability and to increase predation pressure on rodents [35]; these processes are also reflected in the vegetation cover. The effects between vegetation cover and rodent communities are reciprocal, and in this study, we emphasize the effects of vegetation cover on rodent communities, but rodents also affect vegetation cover. Remote sensing monitoring shows a corresponding increase in vegetation cover after rodent eradication [36]. The follow-up studies should focus more on the mechanisms and long-term effects of rodent community–plant community interactions.

## 5. Conclusions

The study conducted the long-term monitoring of rodent communities and vegetation cover and found significant spatial and temporal variability in rodent population density and fatness as well as vegetation cover in Dongting Lake. East Dongting is a key area for rodent control because of its high density and large population fluctuations, while the rodent population in beach fluctuates more than that in farmland, which is an significant source of rodent pests in Dongting. The fatness of rodents in winter was higher than in other seasons at most survey sites, also further threatening agricultural safety in the summer. Population density and fatness were differently related to vegetation cover for different rodent species. The fatness of *M*. *fortis* was more closely related to vegetation cover, but high vegetation cover correlated with *A. agrarius* fatness. The results of the study suggest that fatness is an important factor of rodent community monitoring, as it can reflect the effects of environmental factors such as vegetation on rodent populations and can predict structural changes in rodent communities. At the same time, rodent management in the Dongting Lake area faces significant challenges because of the inverse relationship between population density, fatness, and vegetation cover of *M. fortis* and *A. agrarius*. 

## Figures and Tables

**Table 1:** Correlation between population density and vegetation cover in typical survey sites.

Survey Site	Species	Low		Medium–Low		Medium		Medium–High		High	
		r	*p*	r	*p*	r	*p*	r	*p*	r	*p*
BZZ	*Microtus fortis*	0.464	0.70	0.349	0.185	0.335	0.205	0.502	0.047	−0.468	0.607
	*Apodemus agrarius*	−0.538	0.032	−0.412	0.113	−0.353	0.179	−0.277	0.299	0.378	0.149
CFH	*Microtus fortis*	−0.181	0.444	−0.191	0.420	−0.147	0.536	0.026	0.912	0.062	0.796
	*Apodemus agrarius*	−0.513	0.021	−0.246	0.295	−0.242	0.304	−0.323	0.164	0.386	0.093

**Table 2:** Correlation between fatness and vegetation cover in different seasons.

Survey Site	Species	Season	Low		Medium–Low		Medium		Medium–High		High	
			r	*p*	r	*p*	r	*p*	r	*p*	r	*p*
BZZ	*Microtus fortis*	Spring	−0.573	0.007	−0.276	0.227	−0.158	0.494	−0.113	0.626	0.263	0.250
		Summer	0.052	0.823	0.093	0.688	0.169	0.464	0.271	0.234	−0.231	0.313
		Autumn	−0.205	0.570	−0.864	0.001	−0.894	0.000	−0.834	0.003	0.752	0.012
		Winter	−0.069	0.766	0.039	0.867	0.121	0.602	0.161	0.485	−0.123	0.595
	*Apodemus agrarius*	Spring	−0.187	0.417	0.072	0.757	0.164	0.478	0.098	0.673	−0.052	0.822
		Summer	−0.185	0.421	−0.074	0.749	−0.063	0.785	−0.052	0.824	0.085	0.715
		Autumn	0.116	0.117	0.150	0.517	0.133	0.564	0.283	0.214	−0.162	0.483
		Winter	0.019	0.935	0.064	0.782	0.016	0.945	0.188	0.413	−0.104	0.655
CFH	*Microtus fortis*	Spring	−0.353	0.117	−0.230	0.315	−0.189	0.411	0.029	0.899	0.145	0.532
		Summer	−0.272	0.233	−0.054	0.817	0.046	0.843	−0.012	0.958	0.017	0.941
		Autumn	0.006	0.981	0.308	0.175	0.329	0.145	0.546	0.010	−0.391	0.079
		Winter	−0.239	0.298	−0.060	0.797	−0.058	0.802	0.078	0.736	0.065	0.778
	*Apodemus agrarius*	Spring	0.408	0.066	0.342	0.129	0.369	0.100	0.335	0.137	−0.464	0.034
		Summer	−0.065	0.781	0.185	0.421	0.161	0.486	0.031	0.894	−0.028	0.904
		Autumn	−0.120	0.606	−0.144	0.534	−0.174	0.450	0.058	0.804	0.088	0.704
		Winter	−0.239	0.310	−0.065	0.785	−0.053	0.823	0.082	0.732	0.012	0.960

## Data Availability

The data are contained within this article.

## Supplementary Materials

The following supporting information can be downloaded at: https://www.mdpi.com/article/10.3390/biology14070867/s1, Figure S1: Schematic diagram of rodent community outbreaks in Dongting Lake; Figure S2: Typical habitat landscape of rodents in Dongting Lake beach; Figure S3: Proportion of different rodent species in different seasons at the three main survey sites; Figure S4: Fatness of rodents in Dongting Lake in different years; Figure S5: Vegetation coverage change in Dongting Lake area from 2000 to 2020; Figure S6: Mean value of maximum vegetation coverage in Dongting Lake from 2000 to 2020; Figure S7: Map of vegetation cover change in different seasons from 2000 to 2020 and Table S1: Statistical table of different vegetation coverage ratio in Dongting Lake area from 2000 to 2020; Table S2: Statistical table of the proportion of vegetation coverage at different levels from 2000 to 2020; Table S3: Statistical table of vegetation coverage change in Dongting Lake area from 2000 to 2020; Table S4: Statistical table of vegetation cover ratio of different levels in different seasons from 2000 to 2020.
