# Supplementary material for: Effects of Vegetation Cover on Community Structure of Rodents Based on Long Time Series from Dongting Lake, China
Source: Biology (Basel). 2025 Jul 17;14(7):867. doi: 10.3390/biology14070867 (PMC12292783; doi:10.3390/biology14070867)
Supplement: Supplementary file 1 [file biology-14-00867-s001.zip › biology-3706764-supplementary.pdf]

supplementary material

Effects of Vegetation Cover on Community Structure of Rodents Based on Long Time Series from Dongting Lake, China

Tian Huang [1], Yongcheng Tang [2,3], Yuwen Sun [4], Meiwen Zhang [3], Chen Zhang [3], Yunlin Zhao [2], Xiaoning Nan [4], Zhiyuan Hu [1,*] and Zhenggang Xu [1,2,4,*]

[1] Hunan Engineering Research Center of Ecological Environment Intelligent Monitoring and Disaster,
Prevention and Mitigation Technology in Dongting Lake Region, College of Information and Electronic, Engineering, Hunan City University, Yiyang 413000, China; huangtian@hncu.edu.cn

[2] Hunan Research Center of Engineering Technology for Utilization of Environmental and Resources Plant, Central South University of Forestry and Technology, Changsha 410004, China; tyc2025010101@163.com (Y.T.); rssq198677@163.com (Y.Z.)

[3] Dongting Lake Station for Wetland Ecosystem Research, Institute of Subtropical Agriculture, The Chinese Academy of Sciences, Changsha 410125, China; zhangmw@isa.ac.cn (M.Z.); zhangchenrosetiger@163.com (C.Z.)

[4] College of Forestry, Northwest A&F University, Yangling 712100, China; 2023011663@nwafu.edu.cn (Y.S.); nxn@nwsuaf.edu.cn (X.N.)

* Correspondence: huzhiyuan@hncu.edu.cn (Z.H.); xuzhenggang@nwafu.edu.cn (Z.X.)

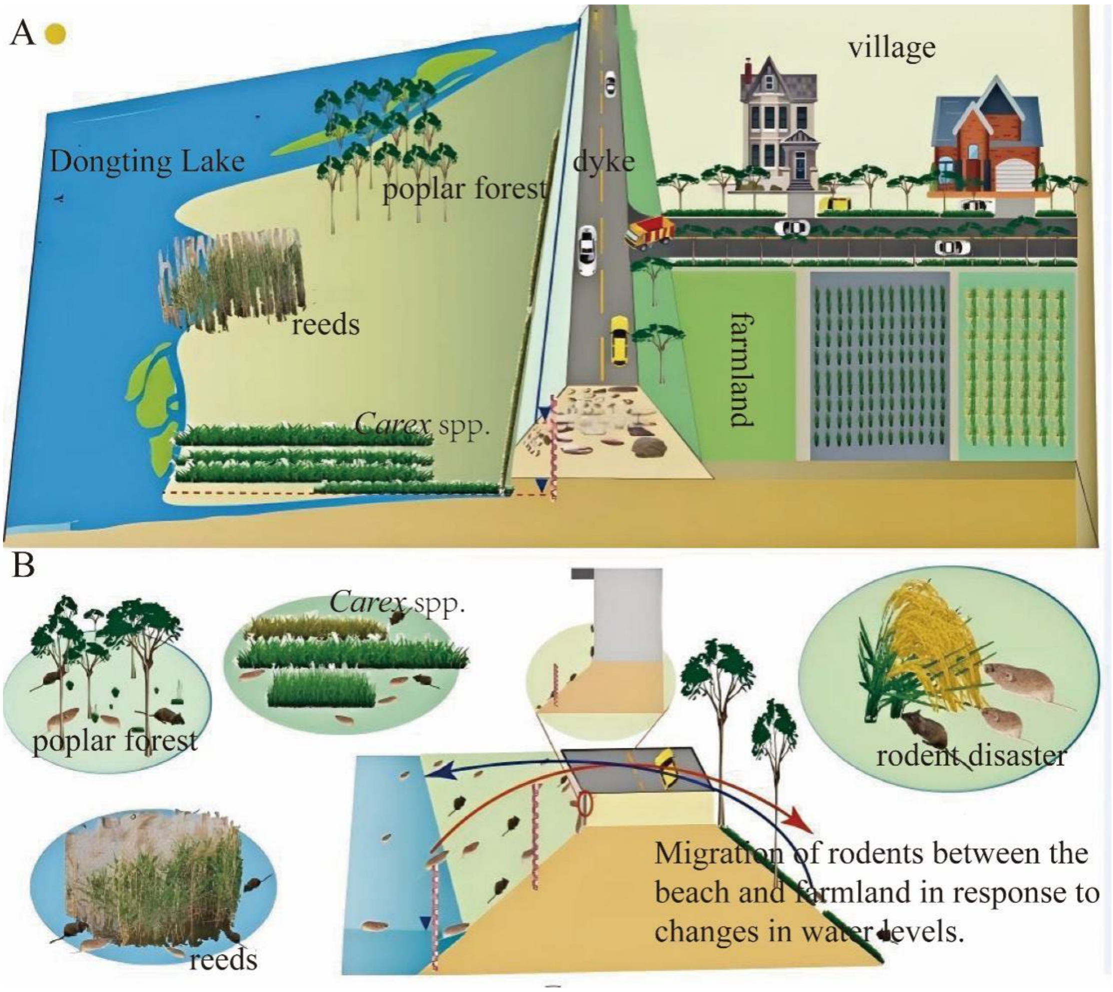

Fig. S1 Schematic diagram of rodent community outbreaks in Dongting Lake.

A: Schematic diagram of the location of beaches and farmland in the Dongting Lake area. B: Water driven migration of rodents between beaches and farmland in Dongting Lake area.

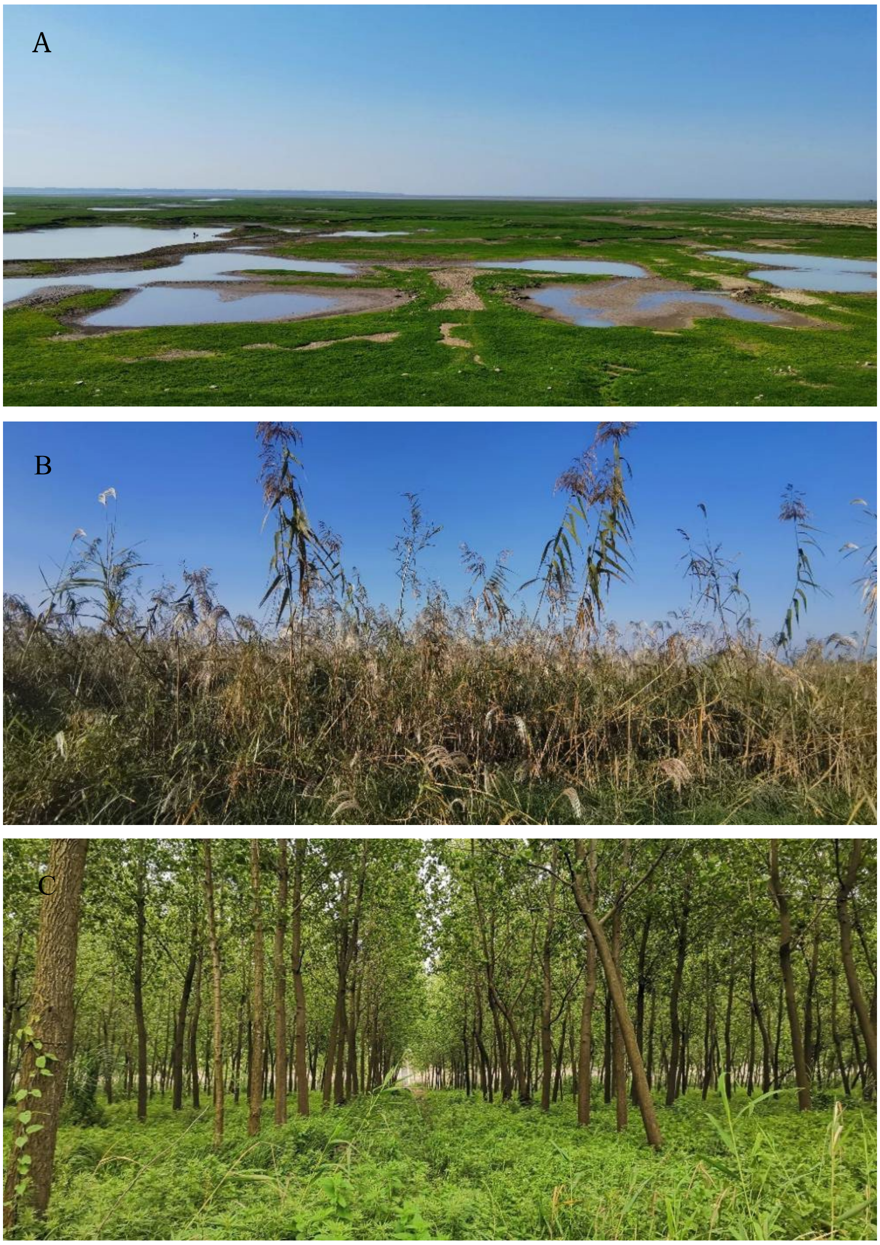

Fig. S2 Typical habitat landscape of rodents in Dongting Lake beach.

A: *Carex spp*.; B: reeds; C: polar forest

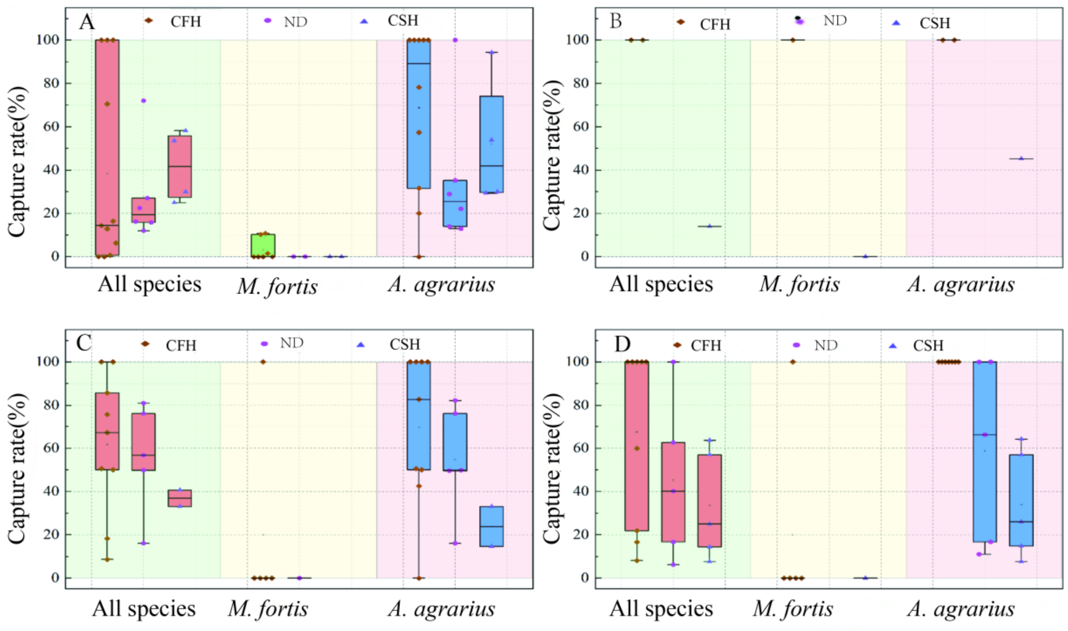

Fig. S3 Proportion of different rodent species in different seasons at the three main survey sites.

A: Spring; B: Summer; C: Autumn; D: Winter

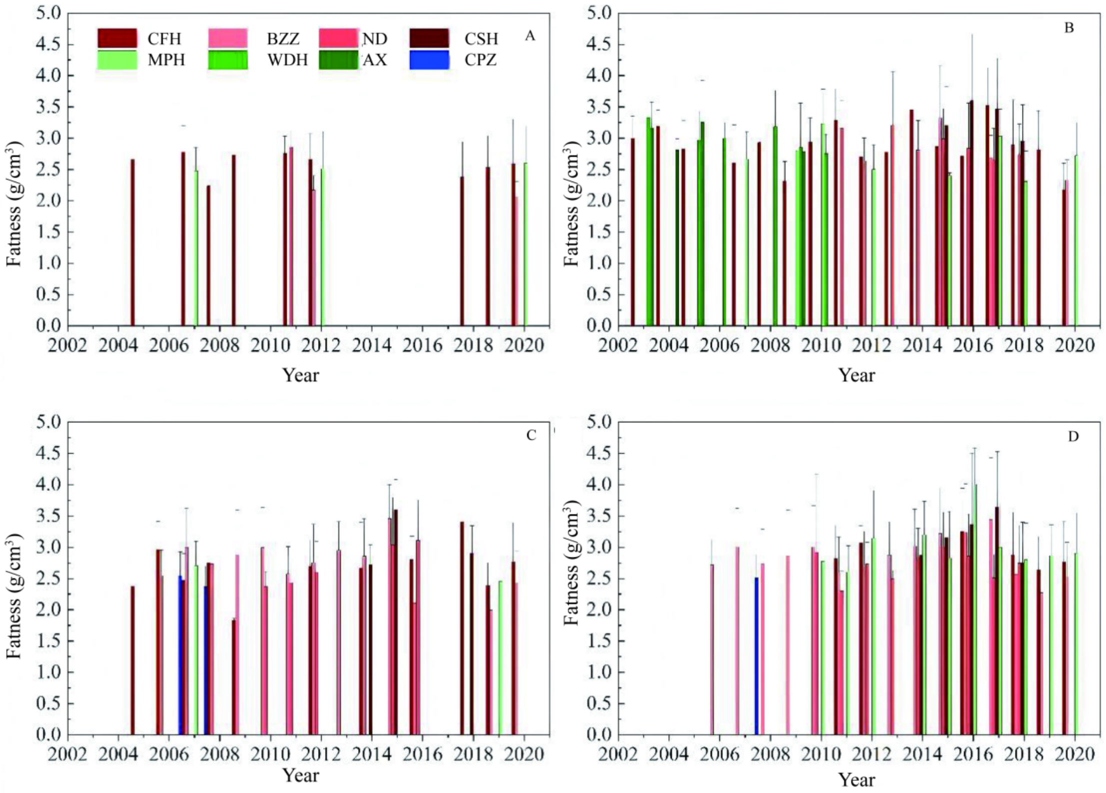

Fig. S4 Fatness of rodents in Dongting Lake in different years.

A: Fatness of *M. fortis* population in farmland; B: Fatness of *A. agrarius* population in farmland;

C: Fatness of *M. fortis* population in beach; D: Fatness of *A. agrarius* population in beach.

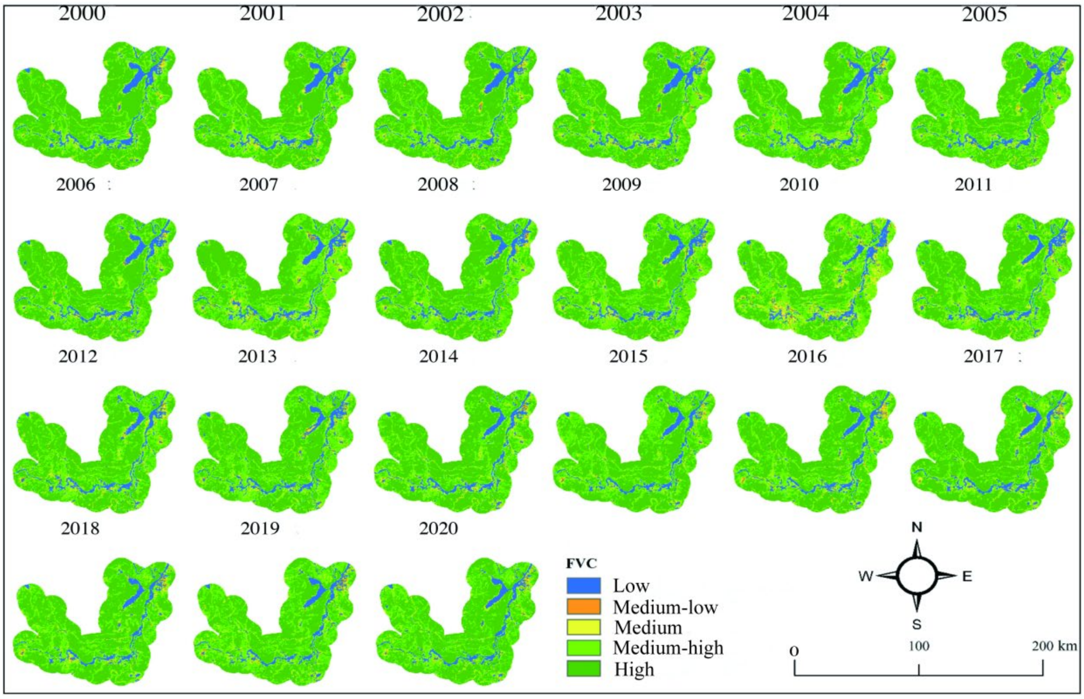

Fig. S5 Vegetation coverage change in Dongting Lake area from 2000 to 2020.

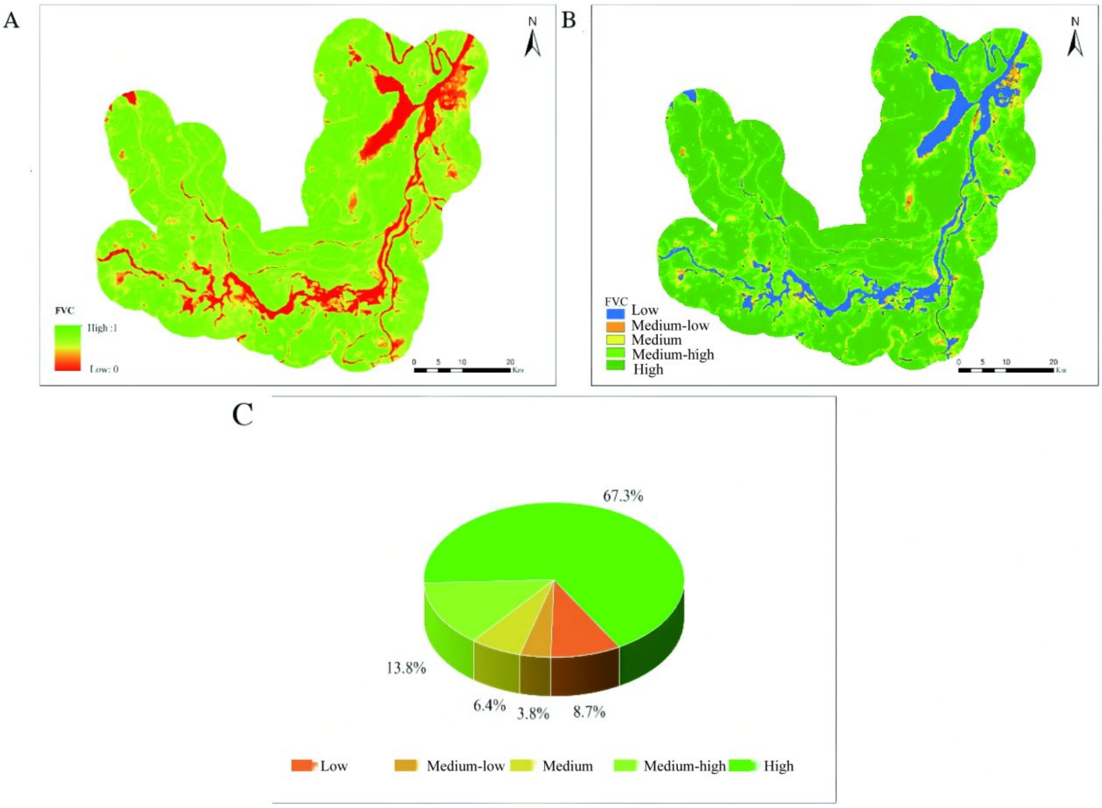

Fig. S6 Mean value of maximum vegetation coverage in Dongting Lake from 2000 to 2020 (A) ,mean classification (B) and Area ratio (C)

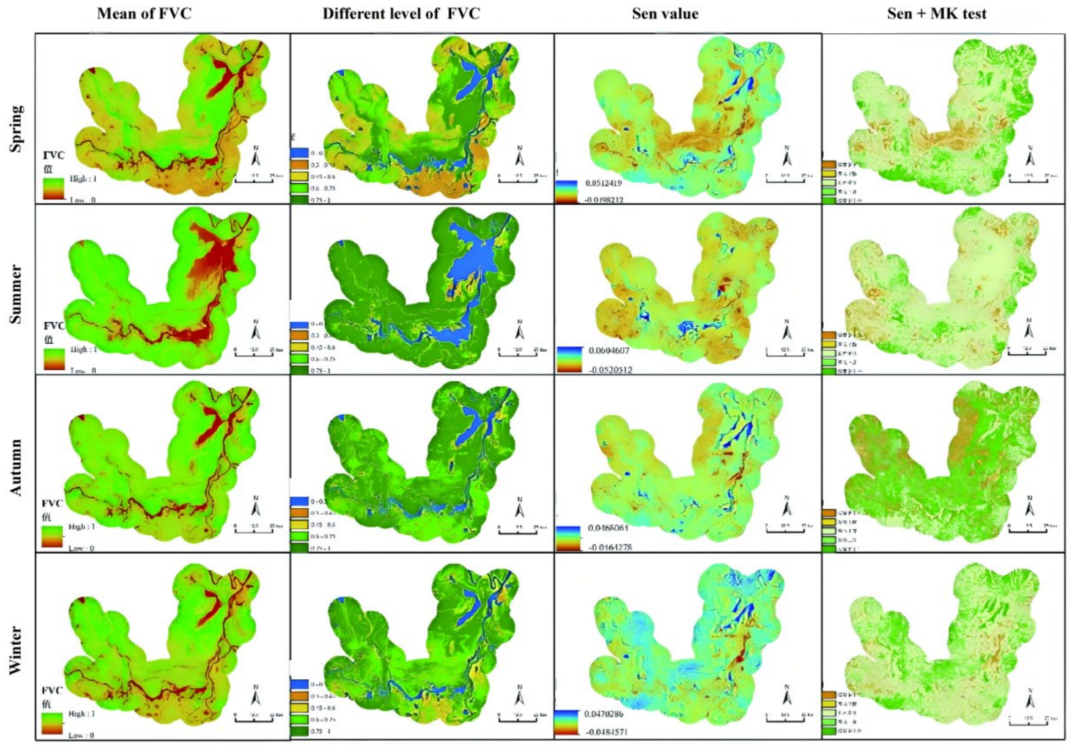

Fig. S7 Map of vegetation cover change in different seasons from 2000 to 2020

Table S1 Statistical table of different vegetation coverage ratio in Dongting Lake area from 2000 to 2020

Year	Low		Medium-low		Medium		Medium-high		High	
	Area (km²)	Percentage (%)	Area (km²)	Percentage (%)	Area (km²)	Percentage (%)	Area (km²)	Percentage (%)	Area (km²)	Percentage (%)
2000	875.06	10.55	289.25	3.49	460.56	5.55	932.75	11.24	5737.88	69.17
2001	843.94	9.73	313.69	3.62	514.25	5.93	1090.69	12.58	5907.81	68.14
2002	887.56	10.24	344.00	3.97	541.13	6.24	1108.00	12.78	5789.13	66.77
2003	897.56	10.24	374.94	4.32	569.50	6.57	1222.50	14.10	5605.56	64.65
2004	906.19	10.45	367.25	4.24	621.50	7.17	1417.75	16.35	5357.00	61.79
2005	846.44	9.76	358.44	4.13	586.69	6.77	1350.75	15.58	5528.13	63.76
2006	852.94	9.84	301.81	3.48	493.13	5.69	1096.63	12.65	5926.00	68.35
2007	885.56	10.21	375.50	4.33	667.19	7.69	1757.56	20.27	4984.69	57.49
2008	820.06	9.46	314.69	3.63	517.88	5.97	1296.88	14.96	5720.94	65.98
2009	838.38	9.67	333.25	3.84	594.19	6.85	1509.81	17.41	5394.69	62.22
2010	870.88	10.04	509.00	5.87	1043.50	12.04	1760.19	20.30	4486.94	51.75
2011	844.94	9.74	337.50	3.89	614.44	7.09	1408.25	16.24	5465.38	63.03
2012	826.81	9.54	338.81	3.91	549.81	6.34	1250.81	14.43	5704.13	65.79
2013	835.25	9.63	330.31	3.81	525.31	6.06	1286.19	14.83	5693.38	65.66
2014	790.63	9.12	313.50	3.62	501.00	5.78	1173.63	13.54	5891.56	67.95
2015	728.00	8.40	253.31	2.92	461.06	5.32	1241.94	14.32	5986.19	69.04
2016	800.13	9.23	315.88	3.64	509.31	5.87	1176.56	13.57	5868.56	67.68
2017	784.13	9.23	304.25	3.51	477.69	5.51	1022.94	11.81	6075.25	70.12
2018	745.75	8.60	274.38	3.16	466.31	5.38	1135.56	13.10	6048.50	69.76
2019	776.06	8.95	306.00	3.53	538.56	6.21	1323.19	15.26	5726.69	66.05
2020	768.94	8.87	289.94	3.34	472.38	5.45	1110.56	12.81	6028.69	69.53

Table S2 Statistical table of the proportion of vegetation coverage at different levels from 2000 to 2020

**Vegetation cover**	**Pixel number**	**Area（km²）**	**Percentage（%）**
Low	12150	759.38	8.74
Medium-low	5289	330.56	3.81
Medium	8859	553.69	6.37
Medium-high	19187	1199.19	13.81
High	93498	5843.63	67.27

Table S3 Statistical table of vegetation coverage change in Dongting Lake area from 2000 to 2020

**FVC changes**	**Pixel number**	**Area（km^2）**	**Percentage(%)**
Very significant rise	5852	365.6	4.21
Significant rise	1973	123.3	1.42
No significant change	113371	7083.5	81.57
Significant decrease	6842	427.3	4.92
Very significant decrease	10945	684.3	7.88

Table S4 Statistical table of vegetation cover ratio of different levels in different seasons from 2000 to 2020

Season	Low		Medium-low		Medium		Medium-high		High	
	Area（km^2）	Percentage（%）	Area（km^2）	Percentage（%）	Area（km^2）	Percentage（%）	Area（km^2）	Percentage（%）	Area（km^2）	Percentage（%）
Spring	936.5	10.8	1271.25	14.65	2371.19	27.33	2060.56	23.75	2035.44	23.46
Summer	1464.63	16.88	370.38	4.27	511.0	5.89	762.56	8.79	5566.38	64.17
Autumn	882.69	10.18	341.13	3.93	649.0	7.48	2401.69	27.69	4400.44	50.73
Winter	937.25	10.8	509.5	5.87	1365.44	15.74	2598.75	29.96	3264.0	37.63
